# Quality of life in patients with primary hyperparathyroidism before and after parathyroidectomy: long term single center experience

**DOI:** 10.1186/s12902-023-01344-z

**Published:** 2023-04-21

**Authors:** T. I. Ionova, D. M. Buzanakov, R. A. Chernikov, S. M. Efremov, I. N. Gladkova, T. P. Nikitina, I. V. Sleptsov, A. V. Zolotoukho, K. A. Bubnov, V. V. Skvortsov, A. A. Vinogradova, V. F. Rusakov

**Affiliations:** 1 Saint-Petersburg State University Hospital, Saint-Petersburg, Russia; 2 Saint-Petersburg State University, Saint-Petersburg, Russia

**Keywords:** Primary hyperparathyroidism, Parathyroidectomy, Quality of life, Symptoms, Long-term follow-up

## Abstract

**Background:**

Primary hyperparathyroidism (PHPT) is a common endocrine disorder caused by a parathyroid tumor or hyperplasia, which is often accompanied with quality of life (QoL) impairment. A parathyroidectomy (PTX) is the preferred standard treatment for PHPT patients. In this single center study we aimed to evaluate the impact of PHPT on patient’s QoL and identify QoL changes at early and long-term follow-up after surgery.

**Methods:**

All the patients underwent routine PTX with the removal of the suspected hyperparathyroid gland(s). Patients filled out generic QoL questionnaire RAND SF-36, specific questionnaire PHPQoL and specific symptom assessment questionnaire PAS upon admission to the hospital before surgery, at 3 months, 12 months and 24 months after surgery.

**Results:**

A total of 92 patients with PHPT (median age was 56 years, 95.7% females) were included in the study. Before PTX patient’s QoL by SF-36 scores was significantly lower as compared to healthy controls (*p* < 0.01). Almost 40% of patients had poor or very poor QoL. The most frequent symptoms by PAS before surgery were as follows: tiredness (97.8% of patients), weakness (94.6%), forgetfulness (94.6%), mood changes (90%), feeling “blue”/depression (88%), joint pains (83.3%), headaches (80.2%), constant irritability (77.2%), bone pains (75%), thirst (70.7%) and trouble getting out of a chair (67.4%). The half of the patients had moderate-to-severe (≥ 40 scores) tiredness, weakness, joint pains, forgetfulness, as well as mood changes. Post-operative QoL changes were analysed in the group of 72 patients. After surgery there was significant improvement in QoL by all scales of SF-36 questionnaire, excluding bodily pain, and the PHPQoL total score (GEE, *p* < 0.01) as compared with their values before surgery. Also severity of tiredness, mood changes, weakness and forgetfulness significantly decreased after surgery as compared to their baseline values (GEE, *p* < 0.05). Decreased mental component of QoL by PHPQoL (OR = 0.927, 95%CI = 0.874–0.984, *p* = 0.013) predicted improved QoL after surgery.

**Conclusions:**

Patients with PHPT demonstrated significantly impaired QoL in physical, psychological and social functioning as well experienced a wide profile of common PHPT symptoms. Successful PTX was accompanied with remarkable QoL improvement and decrease in subjective symptoms for at least 24 months after surgery.

## Background

Primary hyperparathyroidism (PHPT) is a common endocrine disorder caused by a parathyroid tumor or hyperplasia of one or several parathyroid glands, which results in an overproduction of PTH and an overall alteration of bone metabolism and calcium and phosphate levels [1]. Hyperproduction of PTH is accompanied with specific visceral and neurological disorders. Prevalence of PHPT is higher in women and the incidence increases significantly in the elderly [2]. Patients may complain about a wide range of symptoms, such as recurrent nephrolithiasis, bone pain, cognitive alterations, and neuropsychological dysfunction (anxiety, depression, and mood changes), which can be difficult to quantify. These symptoms may evolve for years. Therefore, both “asymptomatic” patients and patients with multiple symptoms can be found [2]. The majority PHPT patients are estimated to be “asymptomatic”. Impaired quality of life (QoL) is considered as a nonclassical manifestation of PHPT [3–5]. Detriments in QoL in PHPT patients have been demonstrated in a number of research [3–7]. Interestingly, till now the data about possible predictors of impaired QoL in PHPT patients are ambiguous and in the most of studies there was found no relationship between QoL and biochemical indices in PHPT [4, 5]. Due to this QoL is complementary to, and cannot be inferred from, the biochemical and other clinical parameters used for patient evaluation [8, 9].

To note, most studies were based on generic questionnaires. Along with the development of disease-specific questionnaires, namely Primary Hyperparathyroidism Quality of Life (PHPQoL) questionnaire and Parathyroidectomy Assessment of Symptoms (PAS), reports on QoL using such tools appeared [10, 11]. In order to study comprehensively the areas of QoL impairment and the extent of detriment in PHPT patients combination of generic and disease- specific QoL tools should be applied. It will help to learn how PHPT affects QoL from the patients' perspective and about their self-perception on the impact of the disease. The combined use of these questionnaires might be helpful to identify differences in QoL between “symptomatic” and “asymptomatic” PHPT patients and to explore patient-reported outcomes (PRO) in patients with different PHPT- disease related characteristics.

A parathyroidectomy (PTX) is the preferred standard treatment for PHPT patients and it is the only curative option with a high success rate [12, 13]. As technical advances in surgery increase, it may not be enough to evaluate procedures with only surgical results. The assessment of the success of surgery from patient’s perspective using PROs is important to provide patient-centered care. It is well known that surgical treatment generally improves QoL in PHPT patients [3, 14–17]. However, according to the results of recent systematic review the data are heterogeneous [18]. Thus, trajectory of QoL changes and symptom elevation at different terms of follow-up after PTX is needed. Sustainability of QoL improvement at long-term follow-up should be confirmed. Furthermore, QoL is not currently routinely assessed preoperatively, and as a result, diminished QoL may be overlooked as an indication for surgery. Identification of preoperative factors associated with the definite QoL improvement is of value for the decision making. Thus, comprehensive research on PROs in PHPT patients before and at different time-points after surgery may highlight the discussion about the feasibility of inclusion of QoL and symptom assessment before surgery and regular PRO assessment at follow-up to monitor patient’s well-being in usual clinical practice.

In this single center study we aimed to evaluate the impact of PHPT on patient’s QoL and identify QoL changes at early and long-term follow-up after surgery.

## Materials and methods

The single-center observational prospective study was carried out from September 2019 to October 2022 in the Saint-Petersburg State University Hospital (St. Petersburg). The inclusion criteria were as follows: 1) confirmed diagnosis of PHPT; 2) age ≥ 18 years old; 3) indication for surgical treatment in accordance with clinical guidelines [15]; 4) written informed consent; 5) the ability of a patient to complete questionnaires. The diagnosis of PHPT was confirmed by clear biochemical diagnosis of PHPT. All other conditions that could mimic the biochemistry of PHPT were ruled out during the preoperative assessment in accordance with clinical guidelines [12]. Patients with both “symptomatic” and “asymptomatic” PHPT were included in the study. “Symptomatic” PHPT is characterized by clinical manifestations – impairment of kidney (nephrolithiasis, nephrocalcinosis, polyuria and polydipsia), skeleton (osteoporosis, fibrocystic osteitis), upper gastrointestinal tract (recurrent ulcers, pancreatitis). “Asymptomatic” type is a confirmed PHPT without skeletal and visceral manifestations with or without nonspecific complaints like fatigue, insomnia, irritation etc. Patients with non-sporadic PHPT were not included in this study. Also patients with significant comorbidities and pronounced mental disorders were excluded as these would likely alter QoL outcomes. To assess comorbidity level the Charlson Comorbidity Index was used [19].

 All the patients underwent routine PTX with the removal of the suspected hyperfunctioning parathyroid glands (HPGs) in the Endocrine Surgery Department of the Clinic. Prior to surgery the following evaluations were performed: serum ionized calcium (Ca^2+^), phosphorus, plasma parathyroid hormone (p-PTH), glomerular filtration rate, vitamin D, creatinine, daily urinary calcium and phosphorus excretion, renal imaging and DEXA or computerized osteodensitometry. Urolithiasis in all the cases was recorded with imaging. Renal insufficiency was defined as eGFR < 60 mL/min. The diagnosis of osteoporosis was made on the basis of the results of DEXA scans.

All operations were made via standard 4-cm Kocher incision by surgeons with extensive experience in endocrine surgery or specializing in parathyroid surgery. In all cases introoperative neuromonitoring of recurrent laryngeal nerves was used. Bilateral neck exploration with the visualization of all fours glands was also performed in cases where multiglandular disease had not been reliably ruled out before the operation. Intraoperative level of p-PTH was not routinely measured. Levels of Ca^2^^+^ and p-PTH were measured 1 day after surgery. Decrease of p-PTH lower high reference level and decrease of Ca^2^^+^ to the target level were achieved for all the patients. In all cases postoperative pathology was also performed. Frozen sections were not used.

When discharged from the hospital after PTX, the patients were given recommendations for taking calcium and active forms of vitamin D under the control of phosphorus-calcium metabolism indicators. The success of surgery was confirmed by normalization of Ca^2^^+^ and p-PTH on the first day after surgery. After the discharge from the hospital the levels of Ca^2+^, p-PTH, 25OHD and other biochemical parameters were controlled on the outpatients’ basis in polyclinics by local endocrinologist or general practitioner in accordance with clinical guidelines [12].

Patients who agreed to complete QoL questionnaires electronically were included in QoL follow-up.

For QoL assessment generic QoL questionnaire RAND SF-36 and specific questionnaire PHPQoL were used. The prevalence and severity of symptoms were analysed on the basis of specific symptom assessment questionnaire for PHPT – PAS.

RAND SF-36 is a widely known generic QoL questionnaire used in healthy subjects and in patients with chronic diseases [20]. The tool is intended for respondents from 14 years of age and consists of 36 questions that form eight scales: physical functioning (PF), role physical functioning (RPF), bodily pain (BP), general health (GH), vitality (V), social functioning (SF), role emotional functioning (REF), and mental health (MH). The time recall period is 4 weeks. After the transforming of the raw data into scaling QoL scores, the results are ranged from 0 to 100 scores for each of the eight scales. The higher the SF-36 questionnaire score, the better the QoL.

The PHPQoL is a disease specific Quality of Life (QoL) questionnaire for use specifically in PHPT patients [10]. It consists of 16 items and has only one dimension. Each item scores on a Likert-type scale ranging from 0 to 4 (always, many times, from time to time, hardly ever, and never). The total score ranges from 0 to 64 and is standardized from 0 (worst QoL) to 100 (best QoL) by applying the formula: (obtained score—minimum score)/(maximum score—minimum score) × 100. The physical component (PC) of QoL (standardized sum of scores on 9 questionnaire questions) and mental component (MC) of QoL (standardized sum of scores on 7 questionnaire questions) are calculated in the same way. The time recall period is 4 weeks. A difference of 9 points for the total score between 2 questionnaire completions was found to be the minimal important difference (MID) perceived by patients in terms of meaningful changes in QoL [8].

The PAS questionnaire was developed to explore 13 items common for PHPT: bone pains, feeling tired easily, mood changes, feeling ‘‘blue’’ or depressed, abdominal pains, feeling weak, feeling irritable, joint pains, being forgetful, difficulty getting out of a chair or car, headaches, itchy skin, and being thirsty [11]. Each item is scored according to the response on a 100-mm visual analogue scale. The PAS score was calculated as the sum of all 13 answers with a maximum possible score of 1300. We analysed severity of each of 13 symptoms of the PAS questionnaire; the PAS total symptom score was not calculated.

Patients filled out the questionnaires several times—upon admission to the hospital before surgery (at baseline), at 3 months, 12 months and 24 months after surgery. At the admission to the hospital patients filled out paper versions of the questionnaires. During the follow-up questionnaires were completed electronically – the research staff contacted patients by phone or email in accordance with study time-points.

The proportion of patients with meaningful QoL improvement after surgery was analyzed on the basis of MID according to PHPQoL total score. Meaningful QoL improvement was considered if at least one MID equal to 9 points by PHPQoL total score after surgery was achieved [8].

To compare QoL in PHPT patients before surgery with healthy controls, a group of the age and sex-matched healthy respondents was created on the basis of earlier conducted normative QoL population study data [21]. By means of the SPSS random number generator, a random sample of 90 out of 2114 respondents who were matched for age and gender to the patient’s group and reported no chronic diseases was formed.

### Statistical analysis

Descriptive statistics for continuous data were presented as number of observations in the group, arithmetic mean and standard deviations, median, range, interquartile range, and 95% confidence interval (CI); for categorical variables, the data were presented as frequencies and percentages. The normal distribution of quantitative variables was determined using Shapiro − Wilk statistical test. For statistical comparisons, we used the Student’s *t*-test, Mann–Whitney U test according to the characteristics of the data, and chi-squared test. To evaluate QoL changes during the follow-up after surgery Generalized Estimating Equations (GEE) were applied. Spearman correlations were used to study the relationship between different baseline variables and QoL scores. Binary logistic regression was used to explore the association between baseline variables and whether the patient had experienced meaningful QoL improvement. To test for multicollinearity, the correlations between independent variables were calculated using the pairwise Spearman correlation coefficient. All the variables which had no strong correlation with each other (Spearman *r* < 0.8) and had a univariate value of *p* < 0.05 were submitted to multivariate regression analysis by the entry method. The results of the binary logistic regression are presented as odds ratio (OR) of the predictors together with 95% CI.

All tests were 2-tailed, with statistical significance level of *p* < 0.05. Statistical analysis was performed using SPSS 23.0 application software package.

## Results

### Patient characteristics

**Fig. 1:**
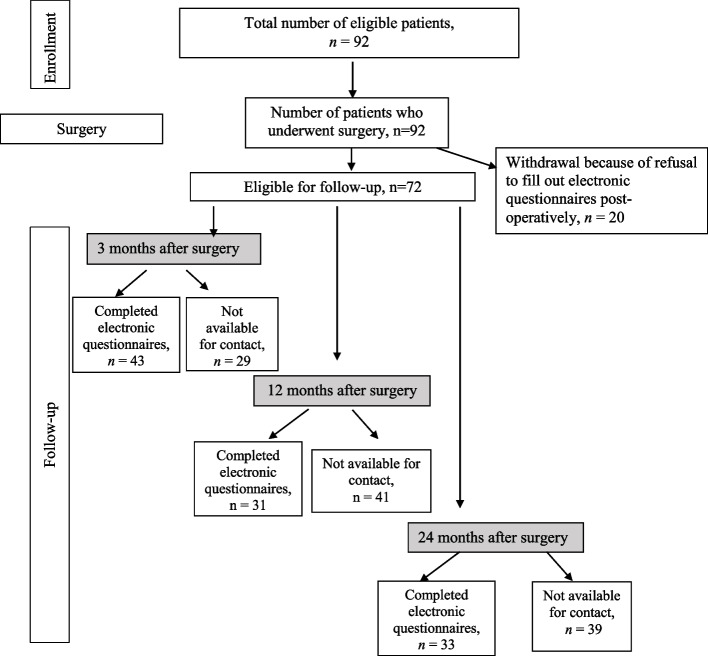
Study flow chart

A total of 92 patients with PHPT were included in the study. Study flow chat is presented in Fig. 1. The decrease of p-PTH lower than upper reference level and the decrease of Ca2 + to the target level on the first day after surgery were achieved for all the patients.

The baseline characteristics of the patients are shown in Table 1. Overall, median age was 56 years, 95.7% were females.

**Table 1:** Baseline characteristics of study patients

**Characteristic**	
Total patients number, n (%)	92 (100%)
**Median age** (range), y.o	56.0 (21–73)
**Number of females/males,** n (%)	88 (95.7)/ 4(4.3)
**Median BMI** (range), kg/m^2^	25.4 (19.3–45.4)
**Median preoperative Ca**^**2+**^(range), mmol/L	1.45 (1.12–1.82)
**Median preoperative p-PTH (**range**),** pmol/L	15.4 (7.2–84.5)
Clinical types of PHPT*, n (%)	
“Asymptomatic”	26 (28.3)
Symptomatic	66 (71.7)
**Hypercalcaemia, n (%)**	
Normocalcemia or mild (Ca^2+^ < 1.49 mmol/L)	58 (63.0)
Moderate (Ca^2+^ = 1.5 -1.79 mmol/L)	31 (33.7)
Severe (Ca^2+^ ≥ 1.8 mmol/L)	3 (3.3)

*BMI* Body Mass Index; Ca^**2+**^ – serum ionized calcium, *p-PTH* Preoperative plasma parathyroid hormone, *PHPT* Primary hyperparathyroidism

Median time from diagnosis to inclusion in the study was 7.7 months (range 0.5–98.3). More than half of the patients had manifest (“symptomatic”) PHPT (71.7%). Moderate or severe hypercalcemia was detected in 37% of patients. Prior to surgery, the median Ca^2+^ value was 1.45 mmol/L (normal range 1.18–1.32), the median preoperative p-PTH value was 15.4 pmol/L (normal range 2.0–8.5 pmol/L). In accordance with the recent WHO classification of parathyroid tumors, all patients were diagnosed with parathyroid adenoma [22]. The size of parathyroid adenoma ranged from 0.8 to 6 cm. Osteoporosis of the spine/hip neck/radius bone was detected in 24 patients (26.4%), urolithiasis – 42 patients (46.7%), while renal insufficiency – 10 patients (11.2%), chronic heart failure – 18 patients (19.6%). Majority of patients had comorbidities (72.8%), including gastrointestinal diseases (66%); cardiovascular pathology (40.3%), endocrine pathology (34.3%), renal diseases (16.4%), joint pathology (9%), respiratory diseases (6%) and others. The median comorbidity index (excluding PHPT complications as urolithiasis and while renal insufficiency) is 1 point (range 0–7).

### QoL in patients with PHPT before surgery

**Fig. 2:**
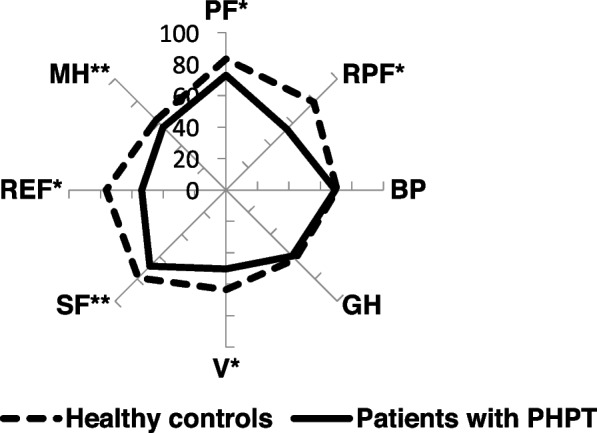
QoL profiles in patients with PHPT before PTX and in healthy controls. *Note.* SF-36 scales – physical functioning (PF), role physical functioning (RPF), bodily pain (BP), general health (GH), vitality (V), social functioning (SF), role emotional functioning (REF), mental health (MH); differences between patients with PHPT and healthy controls (Student's *t*-test for MH and V; Mann–Whitney *U*-test for other scales): *p ≤ 0.001; ^**^p < 0.01

The mean SF-36 profiles for PHPT patients before PTX and for the age- and gender-adjusted healthy controls are presented in Fig. 2. The control group included 86 females and 4 males; mean age ± standard deviation – 53.5 ± 11.5 (range 18–77).

As can be seen from the figure, SF-36 score means in patients with PHPT before surgery are significantly lower than in healthy controls, with the more pronounced decrease of role (physical and emotional) functioning, physical functioning, vitality (*p* < 0.001), social functioning and mental health (*p* < 0.01). The results from QoL assessment using specific PHPQoL questionnaire revealed that the mean baseline total score was 54.4 ± 16 points (range 10.9–95.3 points); the mean PC of QoL was 55.7 ± 17.7 points; the mean MC of QoL was 52.7 ± 16.6 points. The distribution of patients by the PHPQoL total score before surgery was as follows: 0–25 points – 3 patients, 26–50 points – 33 patients, 51–75 points – 45 patients, 76–100 points – 11 patients. According to this distribution, 60.9% of patients exhibited good PHPQoL (corresponds to the range of 51–100 points by PHPQoL total score), 35.9% – poor QoL (26–50 points by PHPQoL total score), and 3.2% of patients – very poor QoL (0–25 points by PHPQoL total score). The most frequent symptoms by PAS in patients with PHPT before surgery were as follows: tiredness (97.8% of patients), weakness (94.6%), forgetfulness (94.6%), mood changes (90%), feeling “blue”/depression (88%), joint pains (83.3%), headaches (80.2%), constant irritability (77.2%), bone pains (75%), thirst (70.7%) and trouble getting out of a chair (67.4%). Less than half of patients experienced abdominal pain (47.3%) and itchy skin (36.7%). Notably, the half of the patients had moderate-to-severe (≥ 40 scores) tiredness, weakness, joint pains, forgetfulness, as well as mood changes. The more symptoms experienced by the patient the worse total PHPQoL score (*r* = -0.45; *p* < 0.001).

Important findings were revealed regarding of relationship between preoperative QoL and type of PHPT. Comparison of QoL scores in patients with “symptomatic” and “asymptomatic” PHPT before surgery revealed that the first ones had significantly lower role physical functioning (46.5 vs 75.0, *p* = 0.002), vitality (47.2 vs 57.7, *p* = 0.023), social functioning (65.3 vs 76.9, *p* = 0.032) and mental health (53.4 vs 64.5, *p* = 0.018) by the SF-36. Also patients with “symptomatic” PHPT had borderline significantly worse PHPQoL total score as compared to patients with “asymptomatic” PHPT (52.4 vs 59.6, *p* = 0.05). They had significantly lower MC score (50.5 vs 58.4, *p* = 0.039); differences by PC were not significant (53.8 vs 60.6, *p* > 0.05). Also patients with “symptomatic” PHPT experienced more severe mood changes and feeling ‘‘blue’’ as compared to patients with “asymptomatic” PHPT (47.3 vs 31.6, *p* = 0.026; 40.5 vs 28.6, *p* = 0.037, consequently). There were no differences in severity for other common PHPT symptoms included in the PAS tool (bone pains, tiredness, abdominal pains, weakness, feeling irritable, joint pains, forgetfulness, difficulty getting out of a chair, headaches, itchy skin, and being thirsty).

The results of correlation analysis for baseline parameters and preoperative QoL by SF-36 scales and PHPQoL total score see below in Table 2.

**Table 2:** Results of correlation analysis for preoperative QoL and baseline parameters

Baseline parameters	SF-36 scales scores								PHPQoL total score
	**PF**	**RPF**	**BP**	**GH**	**V**	**SF**	**REF**	**MH**	
**Age,** yrs	**-.412** **.000**	-.157 .136	**-.232** **.026**	**-.248** **.021**	.038 .728	.106 .313	.093 .385	.131 .218	-.082 .435
**Gender** males vs females	-.165 .116	.010 .921	-.153 .144	-.079 .469	-.110 .304	-.205 .050	-.027 .802	-.088 .408	-.139 .138
**Disease duration,** weeks	-.105 .318	-.063 .551	-.109 .300	-.101 .354	.001 .993	.040 .704	.083 .435	.099 .354	-.104 .324
**Preoperative p-PTH,** pmol/L	-.111 .297	.008 .940	-.162 .127	.105 .342	.004 .984	.074 .487	.103 .339	.055 .614	-.049 .645
**Hypercalcaemia,** mild vs moderate/severe	.042 .729	.127 .293	-.037 .757	.005 .968	.093 .448	.161 .177	.181 .134	.171 .157	.169 .155
**Presence of comorbidities**									
Yes vs no	**-.357** **.000**	**-.290** **.005**	-.082 .440	-.212 .050	**-.330** **.002**	**-.246** **.018**	-.201 .058	**-.245** **.020**	**-.252** **.015**

Values are presented as Spearman’s correlation coefficient with *p* value

*p-PTH* Preoperative plasma parathyroid hormone, *PHPQoL* Primary Hyperparathyroidism Quality of Life questionnaire, SF-36 scales: *PF* Physical functioning, *RPF* Role physical functioning, *BP* Bodily pain, *GH* General health, *V* Vitality, *SF* Social functioning, *REF* Role emotional functioning, *MH* Mental health

There was no significant relationship between preoperative QoL by both SF-36 and PHPQoL and different preoperative PHPT-related variables, such as level of p-PTH, level of hypercalcaemia and disease duration (*p* > 0.05). There were no correlations between preoperative QoL and patients’ gender (*p* > 0.05). Age inversely correlated only with physical functioning (Spearman *r* = -0.412, *p* < 0.001), bodily pain (Spearman *r* = -0.232, *p* = 0.026) and general health (Spearman *r* = -0.248, *p* = 0.021). There was no relationship between age and other SF-36 scales as well as PHPQoL total score (*p* > 0.05). Also we revealed inversed significant correlations between preoperative QoL (by the majority of SF-36 scales and PHPQoL total score score) and the presence of comorbidity.

### QoL in patients with PHPT at early and long-term follow-up after surgery

Patients who completed the questionnaires at least one follow-up time-point were included in the analysis of QoL changes after PTX (*n* = 72).

**Fig. 3:**
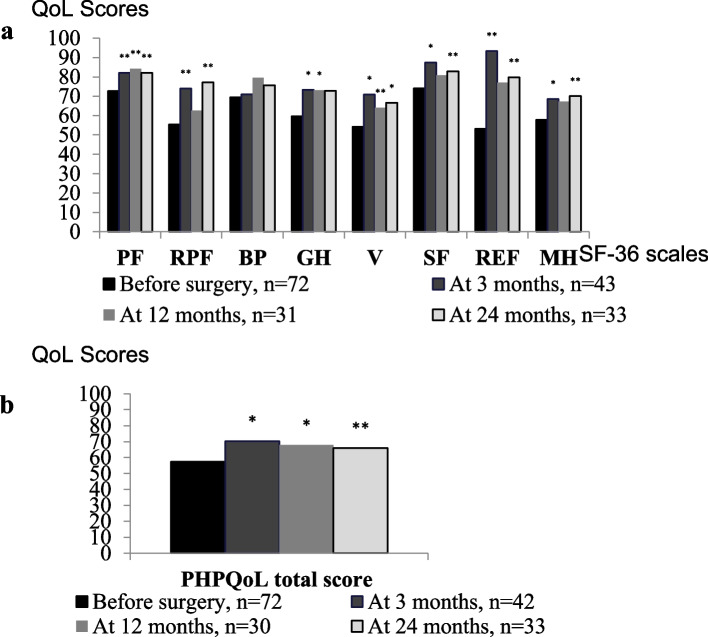
Adjusted means for SF-36 scales (a) and adjusted means for PHPQoL total score (b) in PHPT patients before surgery, at 3, 12 and at 24 months after surgery. Note:*p ≤ 0.001; **p < 0.05

In total, 56% patients withdrew at different time-points of follow-up because of lost contact and withdrew contact. Taking into account CONSORT PRO Guidelines [23] to handle with missing data the analysis of QoL changes in PHPT patients at 3, 12 and at 24 months after PTX as compared with their baseline values was performed using GEE. Adjustment to gender, age, baseline QoL, clinical type of PHPT (“symptomatic” vs “asymptomatic”) and level of hypercalcemia (mild vs moderate/severe) was made. The adjusted mean QoL scores by SF-36 scales and adjusted mean PHPQoL total scores in patients with PHPT before and at different time-points after surgery are shown in Fig. 3.

After surgery there was significant improvement in QoL by all scales of SF-36 questionnaire, excluding bodily pain, as compared with their values before surgery (GEE, *p* < 0.01). The most pronounced positive changes in QoL scores at long-term follow-up after surgery were found for the role physical functioning and role emotional functioning (∆ = 21.9 points, ∆ = 26.6 points, respectively).

There was also a significant increase in the PHPQoL total score (GEE, *p* < 0.001) at different time-points after surgery which corresponds to improved QoL. Adjusted mean of PHPQoL total score increased by 22% at 3 months after surgery, by 18% at 12 months and by 15% at long-term follow-up (57.4 before surgery vs 70.2 at 3 months, 67.8 at 12 months and 66.0 at 24 months). Similar trajectories were identified for changes in PC and MC of PHPQoL. Adjusted means of both PC and MC scores significantly increased at 3 months after surgery, maintained at 12 months and were sustainable at long-term follow-up: 55.2 before surgery vs 71.1 at 3 months, 64.6 at 12 months and 62.7 at 24 months for PC; 52.0 before surgery vs 67.6 at 3 months, 65.0 at 12 months and 64.0 at 24 months for MC (GEE, *p* < 0.001).

**Fig. 4:**
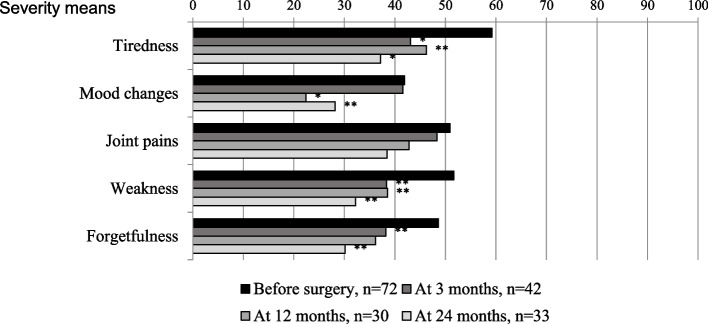
Adjusted means for severity of tiredness, mood changes, joint pains, weakness and forgetfulness in PHPT patients before surgery, at 3, 12 and 24 months after surgery. Note:*p ≤ 0.001; **p < 0.05

Changes in the level of most severe baseline symptoms in patients after PTX were analyzed using GEE with adjustment to the variables mentioned above, including baseline symptom severity instead of baseline QoL value. The adjusted mean severity symptom scores in patients with PHPT before surgery, at 3 months, 12 months and 24 months after surgery are depicted in Fig. 4.

As can be seen from the figure, tiredness, mood changes, weakness and forgetfulness significantly decreased after surgery as compared to their baseline values (GEE, *p* < 0.05).

Meaningful QoL improvement in QoL by PHPQoL (responders) was reported in more than half of operated patients (61.1%). In patients with “asymptomatic” PHPT there were 61.9% QoL responders, in patients with “symptomatic” PHPT – 60.8% QoL responders. In patients with mild hypercalcemia before surgery there were 63.8% QoL responders, in patients with moderate-to-severe hypercalcemia – 56.0% QoL responders. The difference in the number of responders and non-responders in the groups with “symptomatic” and “asymptomatic” PHPT and in the groups with mild and moderate-to-severe baseline hypercalcemia were not significant (χ^2^, *p* > 0.05).

### Factors associated with meaningful QoL improvement in patients with PHPT after surgery

To evaluate the associations between meaningful QoL improvement after surgery and preoperative variables the binary logistic regression analysis was performed. The following variables were analyzed: age, education, pre-surgery level of Ca^2+^, pre-surgery level of p-PTH, “symptomatic” or “asymptomatic” PHPT, duration of the disease, presence of comorbidities, as well as baseline PC and MC of QoL by PHPQoL and baseline level of most severe preoperative symptoms by PAS. The results of the univariate and multivariate analysis predicting whether the patient had experienced meaningful QoL improvement by the PHPQoL total score from baseline over time after surgery are shown in Table 3. In accordance with univariate analysis, pre-surgery level of Ca^2+^, pre-surgery physical and mental components of QoL by PHPQoL, and pre-surgery tiredness, weakness, mood changes and forgetfulness are the significant independent predictors of meaningful QoL improvement after surgery. Taking into account that the high correlations were revealed between baseline level of tiredness and weakness (*r* = 0.8, *p* < 0.001), we included tiredness in the final multivariate regression model as a more pronounced symptom before surgery with slightly higher OR at the univariate level (1.03 for tiredness vs 1.025 for weakness).

**Table 3:** Results of binary logistic regression analysis

Independent variables^a^	Univariate analysis			Multivariate analysis		
	***p-*Value**	**Odds Ratio**	**95% Confidence Interval**	***p-*Value**	**Odds Ratio**	**95% Confidence Interval**
**Age, yrs**	0.313	0.974	0.926–1.025			
**Education**						
Seconday High^**b**^	0.738	0.833	0.287–2.422			
**Disease duration, weeks**	0.49	1.003	0.995–1.012			
**Baseline Ca**^**2+**^**, mmol/L**	**0.049**	0.017	0–0.976	0.119	0.021	0.000–2.693
**Baseline p-PTH, pmol/L**	0.552	0.990	0.960–1.002			
**Clinical types of PHPT:**						
“asymptomatic”						
“symptomatic” ^**b**^	0.929	1.048	0.369–2.98			
**Comorbidities**						
no	0.929	1.048	0.369–2.98			
yes^**b**^						
**Baseline PC by PHPQoL**	**0.032**	0.969	0.942–0.997	0.127	1.043	0.988–1.101
**Baseline MC by PHPQoL**	**0.001**	0.933	0.896–0.972	**0.013**	0.927	0.874–0.984
**Baseline tiredness**	**0.006**	1.03	1.008–1.052	0.185	1.025	0.988–1.063
**Baseline mood changes**	**0.016**	1.024	1.004–1.044	0.975	1.000	0.975–1.025
**Baseline joint pains**	0.203	1.009	0.995–1.022			
**Baseline weakness**^**c**^	**0.004**	1.025	1.008–1.043			
**Baseline forgetfulness**	**0.047**	1.016	1–1.031	0.885	1.002	0.980–1.024

^**a**^Dependent variable—PHPQoL obtained total score increase (reference category – presence of meaningful QoL improvement in ≥ 9 points)

^**b**^Reference category

^c^Baseline weakness was excluded from the final model because of high correlations with baseline tiredness (Spearman *r* = 0.8, *p* < 0.001)

Within the significant final model, of several possible independent patient- and disease-related factors, only preoperative mental component by PHPQoL was significantly predicting meaningful QoL improvement after surgery (Nagelkerke *R*^*2*^ = 0.345; *p* = 0.002). Decreased mental component of QoL by PHPQoL (OR = 0.927, 95%CI = 0.874–0.984, *p* = 0.013) predicted improved QoL after surgery.

## Discussion

Surgery is the only curable treatment in patients with PHPT [12]. Furthermore, it is known that commonly QoL in PHPT is deteriorated [24] and patients may experience diverse and often non-specific symptoms [25–27]. Thus, evaluation of benefits of PTX from patient’s perspective in terms of improving QoL and symptoms is of importance. In addition, as far as the clinical picture of PHPT has changed over the last decades, mainly due to the early detection of hypercalcemia and often occurs as an “asymptomatic” or a mildly “symptomatic” disease [1], the information about the impact of different types of PHPT on QoL might be helpful in decision making. In this single-center prospective observational study we have analyzed comprehensively the impact of PHPT on QoL and investigated the trajectory of changes in PROs during 24 months after successful surgery.

We demonstrated high disease burden in terms of PROs in PHPT patients before surgery using the battery of generic and disease specific QoL questionnaires, namely SF-36 and PHPQoL as well as symptom assessment tool PAS. Patient’s QoL was significantly lower as compared to healthy controls, namely, role physical and role emotional functioning, physical functioning, vitality, social functioning and mental health were decreased. Almost 40% of patients had poor or very poor QoL measured by disease specific PHPQoL questionnaire. This points to the fact that before surgery disease specific areas of QoL in PHPT patients are compromised. Furthermore, symptom profile was analyzed using PAS, the tool developed to assess symptoms of PHPT. The vast majority of patients experienced common PHPT symptoms. Of importance, half of the patients had moderate-to-severe (≥ 40 scores) nonspecific symptoms, such as tiredness, weakness, joint pains, forgetfulness and mood changes. In general, the more symptoms experienced by a patient, the worse his QoL. Impaired QoL in PHPT patients was reported in previous studies [3, 4, 7]. To note, those studies had limitations regarding insufficient patients numbers, lack of control population, insufficient assessment of cognitive areas, and the use of non-specific questionnaires. In our study comprehensive PRO assessment was performed which allowed to illustrate from patient’s perspective full-on disease burden before surgery. The use of generic QoL questionnaire SF-36 which assesses overall health and well-being as well as disease-specific PHPQoL, which measures the impact in two areas important to PHPT patients, physical and emotional/neuropsychological functions, along with symptom assessment tool PAS, sounds worthy to describe patient’s functioning before treatment.

Another important outcome of our study is that using the battery of PRO measures we demonstrated differences in QoL deterioration and symptom burden in patients with “symptomatic” and “asymptomatic” disease. We found that patients with “symptomatic” PHPT had worse QoL and more pronounced psychological problems than patients with “asymptomatic” disease. In general, similar findings have been discussed by other researchers before [3, 4]. However, apart from other studies, we found that differences between “symptomatic” and “asymptomatic” PHPT were mostly in psychosocial domain. Thus, patients with “symptomatic” disease had lower vitality, social functioning, mental health, and role physical functioning by SF-36 as well as lower mental component by PHPQoL as compared to patients with “asymptomatic” PHPT. As for symptoms, we demonstrated that only mood changes and feeling ‘‘blue’’ were more pronounced in patients with “symptomatic” PHPT as compared to “asymptomatic”. The severity of other symptoms was similar in both groups. These data may confirm that patients with “asymptomatic” PHPT have similar symptom profile as patients with symptomatic PHPT, excluding psychological problems, which more pronounced in the latter ones.

For decades, researchers have attempted to understand the factors associated with the reduction and/or maintenance of QoL in PHPT [4, 5, 8, 10, 28, 29]. We found no significant effect of disease-related factors, such as level of preoperative Ca^2^^+^, preoperative p-PTH and disease duration on QoL subcategories. These results confirm the data obtained in the studies by E.M. Ryhanen et al. [4], S.M. Webb et al. [8], and Tsukahara K et al. [9], apart from the results by Ejlsmark-Svensson H. et al. [5], Tzikos G. et al. [28], Mohan B. et al. [29]. Our findings demonstrate that patients with PHPT experience various systemic and neuropsychological symptoms which attribute to reduction of QoL and may be considered as a nonclassical manifestation of PHPT which cannot be inferred from the biochemical and other biomedical parameters used for patient evaluation.

Taking into account that at present QoL assessment has become increasingly important in PHPT patients undergoing PTX to evaluate the effect of surgery, the main goal of our study was to analyze trajectory of changes in generic and disease-specific QoL aspects in PHPT patients at different terms of follow-up after surgery, and to explore the predictive significance of preoperative parameters in terms of meaningful QoL improvement after PTX. In those patients who had successful surgery we demonstrated distinct noticeable positive changes in QoL already at 3 months after PTX. The most remarkable improvement was revealed for psychological and social aspects of QoL measured by generic questionnaire SF-36. Keeping in mind that surgery can exert a placebo effect on these patients, which could explain a very early improvement, and taking into account that some SF-36 scales deteriorated at later time after surgery, we also demonstrated from other side that symptoms continue to improve with time. This finding might be considered as confirmation of rather true improvement at 3 months after PTX then placebo effect. As for PHPT-specific QoL questionnaire we revealed similar trajectory of improvement for both physical and psychological components of QoL after surgery. Also we found that the severity of the most frequent and severe preoperative symptoms – tiredness, weakness, mood changes and forgetfulness decreased significantly at 3 months after surgery. Noteworthy, positive QoL and symptom changes sustained over 24 months after PTX. The results obtained are in accordance with our preliminary data analysis [30] and support valuable changes of PROs after surgery. Improvement of QoL after PTX was reported in other studies [3–7, 24] but only in a few of them there were several study time-points after surgery and long-term follow-up was performed. On the contrary, in our study PROs were measured at 3, 12 and 24 months which allowed to trace the dynamics of QoL and symptoms at different time-points after surgery and to confirm sustained QoL improvement and symptom alleviation at long-term follow-up.

It is remarkable that more than half of the operated patients (61.1%) had clinically meaningful QoL improvement, namely were QoL responders. Here it is worthy to emphasize that small differences in QoL may be statistically significant yet clinically unimportant. The concept of clinically meaningful change in QoL, namely minimal clinically important difference, has been proposed to refer to the smallest difference in a score that is considered to be worthwhile or important [31]. We used the difference of at least 9 points between the baseline and any follow-up time-point PHPQoL total score based on the reports by the PHPQoL developers [8]. Thus, the patients with improvement in PHPQoL total score after surgery by at least 9 points were considered as QoL responders. To note, the proportion of QoL responders was similar in patients with “symptomatic” and “asymptomatic” PHPT. Moreover, no differences in the proportion of QoL responders were observed between patient’s subgroups depending on the level of preoperative hypercalcemia. This finding may be viewed as the evidence of similar benefit of PTX in terms of PROs in patients with “symptomatic” and “asymptomatic” PHPT as well as in patients with different level of preoperative hypercalcemia. Thus, preoperative QoL and its changes after surgery are not dependent on clinical parameters, but are mostly related with neurocognitive problems and psychological symptoms, experienced by the patient due to disease.

The data on predictors of surgery success in PHPT patients are still limited and studies aimed to reveal what preoperative variables may be predictive of QoL improvement along with normalization of disease-related biochemical parameters is worthwhile. To explore the nature of relationship between meaningful QoL improvement and preoperative variables in PHPT patients the regression analysis was applied. The list of baseline independent variables involved those clinically relevant, including type of PHPT, preoperative Ca^2^^+^, p-PTH, disease duration, presence of comorbidities as well as patient’s age and education along with patient-reported outcomes, such as baseline QoL by disease-specific questionnaire and severity of most pronounced PHPT symptoms by PAS. Along with results of E.M. Ryhanen et al. [4], we detected that preoperative QoL is predicting for QoL improvement. On contrary to Ryhanen data, education was not predictive for meaningful QoL improvement in our study. Also all disease-related parameters included in the analysis did not demonstrate their predictive significance for QoL improvement after surgery. Moreover, only mental component of QoL was significantly predicting meaningful QoL improvement after PTX in the final regression model. In other words, the worse psychological condition of patient, the higher probability of meaningful QoL improvement over time after surgery. This finding suggests that the derangement of mental health may be considered as an individual indication for surgery.

Our study has several limitations. First, sufficient number of patients were lost during follow‐up because of electronic administration of PRO measures, which may bias the results. Taking into account commonly high withdrawal rate in prospective QoL studies and taking into consideration CONSORT PRO Guidelines for handling missing data in order to minimize bias we performed GEE for comparisons. Second, the levels of Ca^2^^ +^, p-PTH, 25OHD and other biochemical parameters were not collected by us during long-term follow-up but only 1 day after surgery due to the study design. The control of the above parameters was performed on the outpatients’ basis and these data were not available for investigators. Thus, the disease recurrence during long-term follow up could not be fully excluded for some participants.

Among other study limitations the lack of a control group of non-operated PHPT patients must be mentioned.

In this paper, we examined preoperative PROs in a comprehensive way and analyzed their changes during 24 months after surgery. Future studies should address follow up for more than 24 months after PTX with sufficient number of patients. Also it is of importance to provide comparison of QoL in PHPT patients at long-time follow-up after surgery with healthy controls.

## Conclusion

The results obtained indicated that patients with PHPT demonstrated significantly impaired QoL in physical, psychological and social functioning as well experienced a wide profile of common PHPT symptoms. Half of the patients had moderate-to-severe nonspecific symptoms, such as tiredness, weakness, joint pains, forgetfulness and mood changes. Patients with “asymptomatic” PHPT had better QoL than patients with “symptomatic” disease, meantime they have similar symptom profile as patients with “symptomatic” PHPT, excluding psychological problems, which are more pronounced in the latter ones. Disease-related factors, such as level of preoperative Ca2 + , preoperative p-PTH and disease duration, did not have significant effect on patient’s QoL.

Successful PTX was accompanied with remarkable QoL improvement and decrease in subjective symptoms for at least 24 months after surgery. PROs meaningfully improved at 3 months after PTX and the improvement was sustainable during 24 months. Among independent patient- and disease-related factors only mental QoL component was significantly predicting meaningful QoL improvement after surgery. This finding implies that there is a number of PHPT patients without overt clinical manifestation or prominent hypercalcemia, who also my benefit from PTX, therefore the indications for surgery may be extended.

## Data Availability

The datasets generated and analysed during the current study are not publicly available due to local regulations, but are available from the corresponding author on reasonable request.
